# Sleep duration and mortality in patients with chronic noncommunicable disease: a population-based cohort study

**DOI:** 10.1265/ehpm.23-00249

**Published:** 2024-02-29

**Authors:** Lin Wu, Ruyi Chen, Yuqin Zhang, Huiying Pan, Ying Wang, Xiaowen Wang

**Affiliations:** 1 School of Medicine, Jinhua Polytechnic College, Jinhua 321017, China; 2 Department of Medical Statistics, School of Public Health, Sun Yat-sen University, Guangzhou 510080, China; 3 Center for Public Health and Epidemic Preparedness & Response, Peking University, Beijing 100191, China

**Keywords:** Sleep, Mortality, Cohort, Chronic disease, Noncommunicable disease

## Abstract

**Background:**

Inadequate sleep behaviors may confer a higher risk of premature death, however, evidence in patients with chronic noncommunicable disease (NCD) is scarce. To investigate the relationship between sleep duration and mortality from all-cause and heart diseases in NCD patients from a prospective cohort.

**Methods:**

Totally, 14,171 participants with at least one NCD, including 8275 with hypertension, 7547 with high cholesterol, 4065 with diabetes, and 5815 with chronic renal failure were enrolled from the National Health and Nutrition Examination Survey during 2005–2014. Cox proportional hazard models were performed to estimate the hazard ratio (HR) for sleep duration and mortality after adjusting for potential confounding factors.

**Results:**

After a median follow-up of 9 years, 2514 all-cause deaths were identified. Compared with sleeping 7–8 h/day, sleeping over 8 h/day was significantly associated with a higher risk of all-cause mortality, where the multivariable-HRs were 1.29 (1.11, 1.50) for hypertension, 1.23 (1.01, 1.51) for high cholesterol, 1.44 (1.13, 1.82) for diabetes, and 1.36 (1.10, 1.68) for chronic renal failure. Similar patterns were observed for heart disease mortality. A nonlinear association was detected between sleep duration and mortality in patients with NCD. Age modified the association in patients with hypertension (P-interaction: 0.036). Trouble sleeping modified the association in patients with diabetes (P-interaction: 0.042).

**Conclusions:**

Long sleep duration was associated with higher risks of all-cause and heart disease mortality in patients with chronic NCD. Our findings highlight that improving sleep behaviors may decrease the risk of premature deaths and help to NCD tertiary prevention.

**Supplementary information:**

The online version contains supplementary material available at https://doi.org/10.1265/ehpm.23-00249.

## 1. Background

Noncommunicable diseases (NCD) such as cardiovascular disease (CVD), type 2 diabetes (T2DM), chronic kidney disease (CKD) continue to cause the highest disease burden worldwide. The statistics from the Global Burden of Disease Study 2019 showed that NCD comprised the greatest fraction of deaths, contributing to 74% of total deaths in 2019 [1]. Moreover, total numbers of global deaths from NCD causes increased from 2000 to 2019 by 28%, representing an estimated 33,3 million deaths in 2019. Besides, the impact of NCD grew from causing 47% (or 1.3 billion) of disability-adjusted life-years (DALYs) in 2000 to causing 63% (or 1.6 billion) of DALYs in 2019 [1]. NCD remains a major cause of health loss for all regions of the world. As the number of people with NCD increases, it becomes important to prevent and control these diseases to reduce mortality and disease burden [2].

Exploring the risk factors associated with NCD is vital to eliminate the disease burden, which also has significant implications for clinical guidelines and public health policies in NCD prevention. Some modifiable lifestyle factors have been confirmed to be associated with the prevention of NCD, including tobacco, alcohol, physical inactivity and dietary factors [3, 4]. In recent years, sleeping factors have been implicated in NCD-related morbidity and mortality. Inadequate sleep duration and poor sleep quality were positively associated with NCD multimorbidity [5]. A Mendelian Randomization Study showed a causal association between short (but not long) sleep and CKD [6]. Meta-analyses indicated that divergence from the recommended 7 to 8 hours (7–8 h/day) of sleep was linked to increased risks of cardiovascular events and mortality [7, 8]. It has been supported by accumulating evidence that adherence to a healthy sleep pattern including an adequate sleep duration (7–8 h/day) is associated with lower risks of all-cause and cardiovascular mortality [9–11].

Sleep disorders such as inadequate sleep duration, insomnia, frequent snoring, daytime sleepiness and late chronotype was found to be prevalent among individuals with pre-existing diseases [12–15]. Research on the relationship between sleep and health consequences was scarce among patients with NCD, which might be distinguished between healthy adults and patients. It was suggested that the amount and quality of sleep is crucial in the glycemic control, metabolic function and cardiovascular risk of T2DM patients [16–18]. Evidence from UK biobank showed adherence to a healthy sleep pattern conferred lower risks of incident T2DM and cardiometabolic multimorbidity among individuals with hypertension [19, 20]. However, the discrepancy between sleep duration and clinical outcomes in individuals with CKD deserves further investigation [21–23]. So far, some guidelines have recommended 7–8 h of habitual sleep for primary prevention of CVD or other health benefits in healthy adults and general population [24–27]. To be noted, updates to the Standards of Care in Diabetes—2023 include new recommendations related to sleep health, emphasizing the screening for sleep health in people with diabetes [28]. Nevertheless, there is no consensus regarding the appropriate sleep pattern among patients with chronic NCD.

Therefore, the aim of this present study is to evaluate the associations between sleep duration and the risks of all-cause and heart disease mortality in patients with chronic NCD through a large U.S. prospective cohort. We also performed stratified analyses by age, sex, and other sleeping factors (sleep disorder and trouble sleeping) to test the effect modifications between these associations.

## 2. Methods

### 2.1 Study population

Individuals enrolled in the present study were from the National Health and Nutrition Examination Survey (NHANES), which is a population-based survey for collecting information on the health and nutritional status among community-dwelling U.S. adults. All the data from NHANES could be downloaded publicly at https://wwwn.cdc.gov/nchs/nhanes/. In brief, NHANES contains biological samples of participants’ serum, plasma, and urine across a wide range of measurements. In addition, it contains data from a large number of questionnaires covering a wide range of demographic, socioeconomic, dietary, and health-related questions, as well as a physical examination section that includes physiological measurements, laboratory tests, and other components. The data in NHANES update in the 2-year cycle with multistage probability sampling design. NHANES has got approval from the Institutional Review Board of the National Center for Health Statistics, verifying that all participants have provided informed consent forms.

**Fig. 1:**
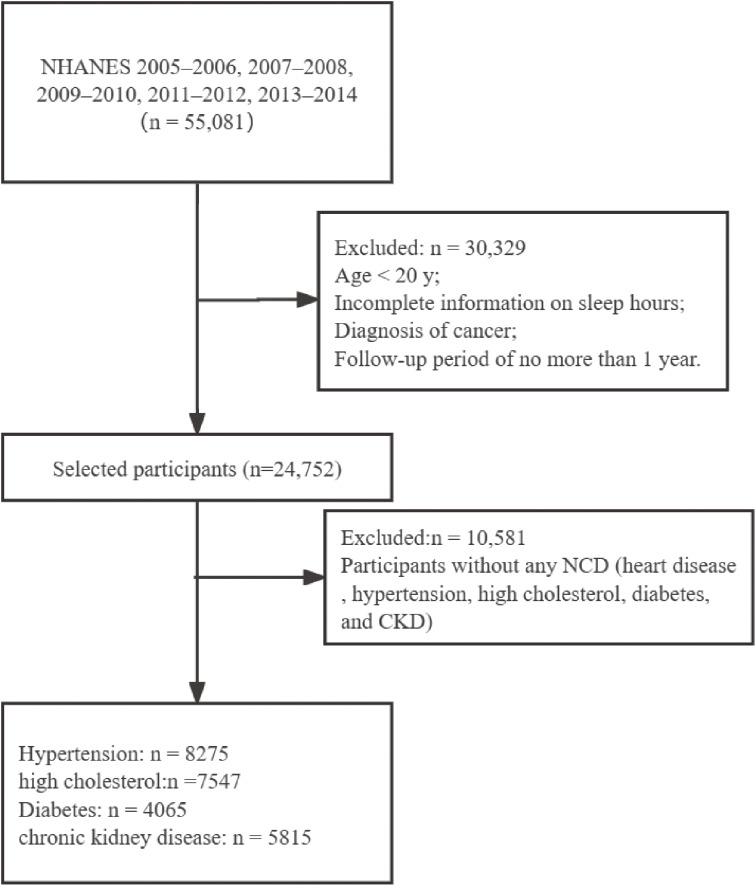
Flow chart of the participants selection.

In this current analysis, a total of 55,081 participants were included from five cycles (2005–2006, 2007–2008, 2009–2010, 2011–2012, 2013–2014). We first excluded those with incomplete information on sleep duration, trouble sleeping and sleep disorder, with diagnosis of cancer, and with a follow-up period of no more than 1 year. Chronic NCD included hypertension, high cholesterol, diabetes, and chronic renal failure. Ascertainment of hypertension and high cholesterol was based on personal interviews. Participants were asked by the survey questions of “Have you ever been told by a doctor or other health professional that you had hypertension, also called high blood pressure?” and “Have you ever been told by a doctor or other health professional that your blood cholesterol level was high?”. Those who answered “yes” were further asked if ever been told to take prescribed medicine. Participants were regarded as cases of diabetes if they were diagnosed to have diabetes by doctors, had fasting blood glucose ≥126 mg/dL or had two-hour oral glucose tolerance ≥200 mg/dL or had HbA1c ≥6.5%. Furthermore, according to the Chronic Kidney Disease-Epidemiology Collaboration equation, glomerular filtration rate (GFR) was calculated [29]: (1) In men, ① serum creatinine ≤0.9 mg/dl: GFR = 141 × (serum creatinine (mg/dl)/0.7)−0.441 × (0.993)age; ② serum creatinine >0.9 mg/dl; GFR = 144 × (serum creatinine (mg/dl)/0.7)−0.441 × (0.993)age. (2) In women, ① serum creatinine ≤0.7 mg/dl: GFR = 144 × (serum creatinine (mg/dl)/0.7)−0.329 × (0.993)age; ② serum creatinine >0.7 mg/dl: GFR = 144 × (serum creatinine (mg/dl)/0.7)−1.209 × (0.993)age. The serum creatinine measurements were obtained from the five cycles and recommended calibrations had been adopted. We defined chronic renal failure as reduced estimated GFR (eGFR) of <30 ml/min/1.73 m^2^. Finally, 14,171 participants with at least one chronic NCD were enrolled in the analysis, including 8275 with hypertension, 7547 with high cholesterol, 4065 with diabetes, and 5815 with chronic renal failure (Fig. 1).

### 2.2 Assessment of sleep and covariates

Each participant was asked about routinely sleep hours from a question of “How much sleep do you get (hours)?”. Sleep duration was categorized as “3–6 h/day, “7–8 h/day (reference)”, and “>8 h/day”. Participants were also interviewed by questions of “Have you ever told a doctor or other health professional that you have trouble sleeping?” and “Have you ever been told by a doctor or other health professional that you have a sleep disorder?”, both of which were categorized as “Yes” or “No”. These questions were asked in the home by using the Computer-assisted Personal Interview system. Covariates included demographic and socioeconomic factors (sex, age, race, education level, and income), lifestyles, and physical health status (hypertension, high cholesterol, diabetes and heart disease).

### 2.3 Follow-up and outcomes

Outcomes for our study were mortality from all cause and heart diseases. Deaths data were obtained from longitudinal Medicare and mortality data with the National Death Index until the date of 31 December 2019. According to the International Classification of Diseases, 10th edition (ICD-10). The all-cause mortality and mortality from heart diseases (I00–I09, I11, I13, I20–I51) were evaluated. Person-year was estimated for patients from the start of the study until to date of death or 31 December 2019, whichever came first.

### 2.4 Statistical analysis

We calculate the appropriate statistics to be representative of U.S. adults by taking into account the survey design such as sampling, stratification, and clustering in NHANES. Baseline characteristics of the patients were described across different sleep duration. Categorical variables were shown as numbers (%), and the differences among groups were tested by Rao-Scott chi-squared (χ2) test. Continuous variables were expressed as mean ± standard deviation, which were tested by analysis of variance with adjustment for sampling weights. Survey-weighted multivariable Cox proportional hazard models were used to estimate the hazard ratios (HRs) with 95% confidence intervals (CIs) of mortality by sleep duration among participants with chronic NCD. The proportional hazards assumption was tested by comparing models with and without a multiplicative interaction term between sleep and follow-up duration. There was no evidence of assumption violations in every model (P > 0.05 for all tests). Model 1 was adjusted for sex (men vs. women) and age (continuous). Model 2 was further adjusted for ethnicity (Mexican American, other Hispanic, Non-Hispanic White, Non-Hispanic Black), BMI (underweight: BMI < 18.5; normal weight: 18.5 ≤ BMI < 25; overweight: 25 ≤ BMI < 30; obesity: BMI ≥ 30), income (family income-to-poverty ratio ≤1.0 vs. >1.0), education level (high school or below, vs. college or above), smoking status (yes or no), alcohol intake (yes or no), moderate physical activity (yes or no), hypertension (yes or no), high cholesterol (yes or no), diabetes (yes or no), and heart disease (yes or no). Model 3 was based on model 2 and additionally adjusted for trouble sleeping (yes or no) and sleep disorder (yes or no). Missing values were treated as separate categories. Restricted cubic splines were constructed for illustrating the dose–response association of sleep duration with all-cause mortality in patients with chronic NCD (3 knots at the 10.0th, 50.0th, and 90.0th percentiles). Subgroup analyses were used to investigate the associations in patients of different sex, age, and sleep factors (trouble sleeping and sleep disorder). The P-value of interaction was estimated by adding a cross-product term between dichotomous risk factors (0 and 1) and the three-category variable of sleep duration (0–2). Sensitivity analyses were also conducted by using sleep hours of 6 h/day as reference, by using albuminuria as CKD outcomes, by excluding patients with mortality events during the first 3 years of follow-up, and by including participants without any NCD (heart disease, hypertension, high cholesterol, diabetes, and CKD in this study). Albuminuria was defined as a urine albumin-to-creatinine ratio ≥30 mg/g. All statistical analyses were performed by using SAS version 9.4 TS Level 1 M6 software (SAS Institute Inc., Cary, NC, United States).

## 3. Results

Table 1 shows the baseline characteristics of patients with at least one chronic NCD according to sleep durations. Compared with those who had a sleep duration of 7–8 h/day, patients who slept 3–6 h/day were younger, less educated, more likely to have obesity, hypertension, diabetes, and higher eGFR, but less likely to have trouble sleeping and sleep disorder. Patients who slept over 8 hours were more often women, older, less educated, more likely to have obesity, hypertension, diabetes, and lower eGFR (Table 1).

**Table 1:** Characteristics of participants with at least one chronic NCDs at baseline.

	**Sleep hours**			**P**
	**3–6 h/day** **(n = 5809)**	**7–8 h/day** **(n = 7278)**	**>8 h/day** **(n = 1084)**	
**Sex**				<0.001
Men	3023 (52.0)	3818 (52.5)	501 (46.2)	
Women	2786 (48.0)	3460 (47.5)	583 (53.8)	
**Age, yr**	53.0 ± 15.4	54.6 ± 16.2	58.4 ± 18.0	<0.001
**Race**				<0.001
Mexican American	777 (13.4)	1134 (15.6)	152 (14.0)	
Other Hispanic	567 (9.8)	666 (9.2)	85 (7.8)	
Non-Hispanic White	2070 (35.6)	3346 (46.0)	526 (48.5)	
Non-Hispanic Black	1865 (32.1)	1462 (20.1)	248 (22.9)	
Other Race - Including Multi-Racial	530 (9.1)	670 (9.2)	73 (6.7)	
**Education**				<0.001
High School or below	3055 (52.6)	3624 (49.9)	644 (59.6)	
College or above	2749 (47.4)	3643 (50.1)	437 (40.4)	
**Family income-to-poverty ratio**				<0.001
≤1.0	1663 (28.6)	1933 (26.6)	364 (33.6)	
>1.0	4146 (71.4)	5345 (73.4)	720 (66.4)	
**BMI**				<0.001
Underweight	421 (7.3)	637 (8.8)	103 (9.5)	
Normal weight	1040 (17.9)	1505 (20.7)	215 (19.8)	
Overweight	1748 (30.1)	2387 (32.8)	321 (29.6)	
Obesity	2600 (44.8)	2749 (37.8)	445 (41.1)	
**Drinking**				<0.001
Non-drinker	1461 (29.5)	1846 (30.1)	311 (33.4)	
Drinker	3500 (70.6)	4292 (69.9)	619 (66.6)	
**Hypertension**				
No	2352 (40.5)	3449 (47.4)	447 (41.2)	
Yes	3457 (59.5)	3829 (52.6)	637 (58.8)	
**High cholesterol**				0.302
No	2846 (49.0)	3471 (47.7)	531 (49.0)	
Yes	2963 (51.0)	3807 (52.3)	553 (51.0)	
**Diabetes**				<0.001
No	4175 (71.9)	5402 (74.2)	732 (67.5)	
Yes	1634 (28.1)	1876 (25.8)	352 (32.5)	<0.001
**eGFR, mL/min/1.73 m^2^**	82.3 ± 26.8	80.7 ± 26.8	77.5 ± 30.1	<0.001
**Trouble sleeping**				<0.001
No	2199 (37.9)	1476 (20.3)	230 (21.2)	
Yes	3610 (62.1)	5802 (79.7)	854 (78.8)	
**Sleep disorder**				<0.001
No	768 (13.2)	572 (7.9)	96 (8.9)	
Yes	5041 (86.8)	6706 (92.1)	988 (91.1)	

Data are mean ± standard deviation or number (%)

NCD, noncommunicable disease; BMI, body mass index; eGFR, estimated glomerular filtration rate.

**Fig. 2:**
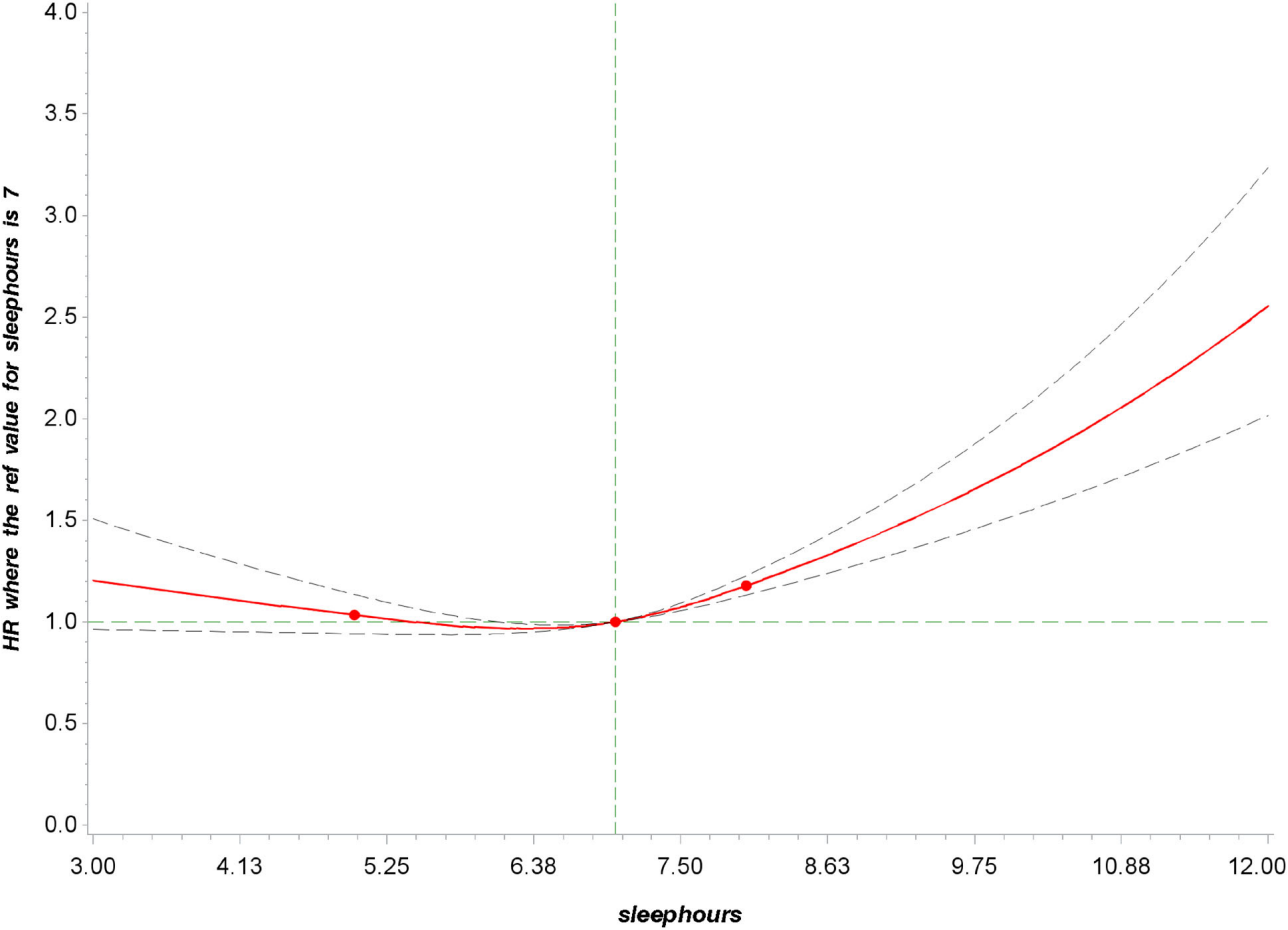
Dose–response relationship between sleep duration and all-cause mortality in participants with at least one chronic NCD. P overall association < 0.001; P nonlinear association < 0.001. Restricted cubic splines were constructed with four knots located at the 10th, 50th, and 95th percentiles of the exposure. Adjusted hazard ratios (95% CI) were calculated with adjustment for age, sex, ethnicity, body mass index, income, education level, smoking status, alcohol intake, moderate physical activity, hypertension, high cholesterol, diabetes, and heart disease.

During a median follow-up of 9 years, we identified 2514 all-cause deaths and 850 heart disease-caused deaths, including 1749 all-cause (597 heart disease-caused) deaths in patients with hypertension, 1235 (432 heart disease-caused) deaths in patients with high cholesterol, 1020 (351 heart disease-caused) deaths in patients with diabetes, and 1440 (498 heart disease-caused) deaths in patients with chronic renal failure. The associations of sleep duration with all-cause and heart disease mortality in NCD patients are illustrated in Table 2. After adjustment for age, sex, ethnicity, body mass index, income, education level, smoking status, alcohol intake, moderate physical activity, hypertension, high cholesterol, diabetes, and heart disease, compared with sleeping 7–8 h/day, sleeping 3–6 h/day was not significantly associated with all-cause mortality, where the HRs (95%CIs) were 1.00 (0.86, 1.17) for hypertension, 1.06 (0.88, 1.26) for high cholesterol, 1.06 (0.85, 1.32) for diabetes, and 0.99 (0.83, 1.18) for chronic renal failure. Patients sleeping >8 h/day had a higher risk of all-cause mortality, where the HRs (95%CIs) were 1.29 (1.10, 1.50) for hypertension, 1.24 (1.01, 1.51) for high cholesterol, 1.43 (1.13, 1.81) for diabetes, and 1.36 (1.10, 1.69) for chronic renal failure. Further adjustment for trouble sleeping and sleep disorder (Model 3) yielded similar results, with HRs of 1.29 (1.11, 1.50), 1.23 (1.01, 1.51), 1.44 (1.13, 1.82), and 1.36 (1.10, 1.68) among participants sleeping more than 8 h/day in comparison to those with 7–8 h/day in patients with hypertension, high cholesterol, diabetes, and chronic renal failure, respectively. The results for heart disease mortality were similar to those for all-cause mortality in relation to sleep duration. Significant association was detected between long sleep duration and heart disease mortality, where the fully-adjusted HRs (95%CIs) were 1.78 (1.32, 2.40) for hypertension, 1.65 (1.12, 2.42) for high cholesterol, 2.34 (1.65, 3.30) for diabetes, and 1.55 (1.04, 2.31) for chronic renal failure. In the restricted cubic spline analysis, a nonlinear association was found between sleep duration and all-cause mortality in patients with chronic NCD (P-overall association < 0.001; P-nonlinear association < 0.001) (Fig. 2 and Supplementary Fig. 1–4).

**Table 2:** Hazard ratios (HRs) of mortality in relation to sleep duration in patients with NCD.

	**All-cause mortality**			**Heart disease mortality**		

	**3–6 h/day**	**7–8 h/day**	**>8 h/day**	**3–6 h/day**	**7–8 h/day**	**>8 h/day**
**Hypertension (n = 8275)**						
Person-year	32457.8	35309.5	5601.0	32457.8	35309.5	5601.0
No. of cases	660	842	247	211	285	101
HR, model 1	1.12 (0.98, 1.28)	1.00	**1.48 (1.26, 1.73)**	1.00 (0.75, 1.32)	1.00	**1.97 (1.48, 2.61)**
HR, model 2	1.00 (0.86, 1.17)	1.00	**1.29 (1.10, 1.50)**	0.91 (0.66, 1.24)	1.00	**1.79 (1.33, 2.41)**
HR, model 3	1.00 (0.86, 1.17)	1.00	**1.29 (1.11, 1.50)**	0.92 (0.68, 1.24)	1.00	**1.78 (1.32, 2.40)**

**High cholesterol (n = 7547)**						
Person-year	27856.1	35562.8	4988.7	27856.1	35562.8	4988.7
No. of cases	451	618	166	159	214	59
HR, model 1	1.24 (1.05, 1.46)	1.00	**1.42 (1.16, 1.75)**	1.34 (0.99, 1.82)	1.00	**1.81 (1.23, 2.66)**
HR, model 2	1.06 (0.88, 1.26)	1.00	**1.24 (1.01, 1.51)**	1.20 (0.86, 1.68)	1.00	**1.66 (1.14, 2.43)**
HR, model 3	1.05 (0.88, 1.26)	1.00	**1.23 (1.01, 1.51)**	1.22 (0.88, 1.70)	1.00	**1.65 (1.12, 2.42)**

**Diabetes (n = 4065)**						
Person-year	15123.5	16983.2	3031.8	15123.5	16983.2	3031.8
No. of cases	386	480	154	140	146	65
HR, model 1	1.13 (0.92, 1.38)	1.00	**1.49 (1.17, 1.90)**	1.19 (0.86, 1.64)	1.00	**2.42 (1.71, 3.43)**
HR, model 2	1.06 (0.85, 1.32)	1.00	**1.43 (1.13, 1.81)**	1.12 (0.78, 1.61)	1.00	**2.37 (1.69, 3.32)**
HR, model 3	1.06 (0.86, 1.31)	1.00	**1.44 (1.13, 1.82)**	1.18 (0.84, 1.67)	1.00	**2.34 (1.65, 3.30)**

**Chronic renal failure (n = 5815)**						
Person-year	19859.0	27069.2	4203.7	19859.0	27069.2	4203.7
No. of cases	479	751	210	159	262	77
HR, model 1	1.12 (0.97, 1.28)	1.00	**1.49 (1.23, 1.81)**	1.03 (0.79, 1.35)	1.00	**1.56 (1.07, 2.28)**
HR, model 2	0.99 (0.83, 1.18)	1.00	**1.36 (1.10, 1.69)**	0.93 (0.67, 1.28)	1.00	**1.55 (1.03, 2.32)**
HR, model 3	1.00 (0.84, 1.19)	1.00	**1.36 (1.10, 1.68)**	0.94 (0.68, 1.29)	1.00	**1.55 (1.04, 2.31)**

NCD, noncommunicable disease.

Model 1 was the Cox proportional hazard model adjusted for age and sex.

Model 2 was based on model 1 and further adjusted for ethnicity, body mass index, income, education level, smoking status, alcohol intake, moderate physical activity, hypertension, high cholesterol, diabetes, and heart disease.

Model 3 was based on model 2 and additionally adjusted for trouble sleeping and sleep disorder.

The subgroup analyses for associations of sleep duration with all-cause mortality risk stratified by sex, age, trouble sleeping, and sleep disorder are shown in Supplementary Table 1, Table 3, Table 4, and Supplementary Table 2, respectively. Similar patterns of associations were observed, although some of the associations were not significant. Interaction analysis showed that sex did not modify the association between sleep duration and all-cause mortality (all P-interactions > 0.10) (Supplementary Table 1). Age modified the association between sleep duration and all-cause mortality risk in patients with hypertension (P-interaction = 0.036), where a significant higher risk was observed for sleeping over 8 h/day in comparison to 7–8 h/day in those aged ≥65 years, with HR of 1.23 (1.01, 1.49) in the fully-adjusted model (Table 3). Trouble sleeping modified the association between sleep duration and all-cause mortality risk in patients with diabetes (P-interaction = 0.042). Compared with those who slept 7–8 h/day, the risk of mortality for sleeping more than 8 h/day was significantly higher in those having trouble sleeping (HR = 1.62 [1.00, 2.65]) than having no trouble sleeping (HR = 1.38 [1.07, 1.78]) in diabetic patients (Table 4). Sleep disorder did not modify the association between sleep duration and all-cause mortality in NCD patients (all P-interactions > 0.10) (Supplementary Table 2). The sensitivity analyses were conducted to test the robustness of the results (Supplementary Table 3–5). First, by using the 6 h/day as cutoff points and reference, the results are in line with previous results that sleeping over 8 h/day was significantly associated with a higher risk of all-cause mortality (Supplementary Table 3). Second, when using albuminuria as another kidney disease status at baseline, the multivariable HR of sleeping over 8 h/day in Model 3 for all-cause mortality was 1.31 (1.01, 1.71). Third, after excluding patients with mortality events during the first 3 years of follow-up, a similar pattern of association of sleep duration with all-cause mortality was observed (Supplementary Table 4). Last, analyses in participants without any NCD also supported that divergence from 7–8 h/day of sleep was linked to a higher risk of mortality (Supplementary Table 5 and Supplementary Fig. 5).

**Table 3:** Age-specific hazard ratios (HRs) for all-cause mortality according to sleep duration.

	**<65 years**			**≥65 years**		
	**3–6 h/day**	**7–8 h/day**	**>8 h/day**	**3–6 h/day**	**7–8 h/day**	**>8 h/day**
**Hypertension**						
Person-year	23019.1	22134.2	2700.3	9438.8	13175.3	2900.7
No. of cases	238	206	48	422	636	199
HR, model 1	1.26 (0.98, 1.63)	1.00	1.90 (1.27, 2.83)	1.00 (0.84, 1.18)	1.00	**1.33 (1.11, 1.59)**
HR, model 2	0.96 (0.72, 1.27)	1.00	1.31 (0.87, 1.98)	0.99 (0.81, 1.19)	1.00	**1.22 (1.00, 1.48)**
HR, model 3	0.94 (0.71, 1.25)	1.00	1.31 (0.87, 1.99)	0.97 (0.80, 1.17)	1.00	**1.23 (1.01, 1.49)**

**High cholesterol**						
Person-year	20637.8	24143.6	2686.5	7218.3	11419.2	2302.2
No. of cases	168	156	33	283	462	133
HR, model 1	1.45 (1.05, 2.02)	1.00	1.74 (1.04, 2.91)	1.28 (1.02, 1.61)	1.00	1.28 (1.02, 1.61)
HR, model 2	1.01 (0.72, 1.43)	1.00	1.16 (0.69, 1.94)	1.18 (0.95, 1.47)	1.00	1.18 (0.95, 1.47)
HR, model 3	1.03 (0.73, 1.45)	1.00	1.16 (0.69, 1.95)	1.17 (0.94, 1.46)	1.00	1.17 (0.94, 1.46)

**Diabetes**						
Person-year	10292.5	10521.3	1482.6	4831.0	6462.0	1549.2
No. of cases	146	124	39	240	356	115
HR, model 1	1.42 (1.00, 2.03)	1.00	2.43 (1.37, 4.33)	0.97 (0.77, 1.23)	1.00	1.23 (0.97, 1.57)
HR, model 2	1.13 (0.78, 1.64)	1.00	1.64 (0.90, 2.99)	0.95 (0.71, 1.29)	1.00	1.25 (0.99, 1.59)
HR, model 3	1.19 (0.83, 1.69)	1.00	1.69 (0.94, 3.05)	0.94 (0.70, 1.25)	1.00	1.27 (1.00, 1.62)

**Chronic renal failure**						
Person-year	13752.3	17311.8	1967.6	6105.7	9757.4	2236.1
No. of cases	124	141	27	355	610	183
HR, model 1	1.40 (1.05, 1.87)	1.00	2.60 (1.47, 4.59)	1.04 (0.89, 1.22)	1.00	1.32 (1.06, 1.63)
HR, model 2	1.09 (0.78, 1.54)	1.00	1.74 (0.94, 3.26)	0.91 (0.75, 1.11)	1.00	1.22 (0.97, 1.54)
HR, model 3	1.11 (0.78, 1.58)	1.00	1.74 (0.93, 3.26)	0.92 (0.76, 1.11)	1.00	1.22 (0.97, 1.54)

Model 1 was the Cox proportional hazard model adjusted for age and sex.

Model 2 was based on model 1 and further adjusted for ethnicity, body mass index, income, education level, smoking status, alcohol intake, moderate physical activity, hypertension, high cholesterol, diabetes, and heart disease.

Model 3 was based on model 2 and additionally adjusted for trouble sleeping and sleep disorder.

P-interaction between sleep hour and age = **0.036 for hypertension**, 0.235 for high cholesterol, 0.044 for diabetes, and 0.009 for chronic renal failure.

**Table 4:** Hazard ratios (HRs) for all-cause mortality according to sleep duration, stratified by trouble sleeping.

	**No trouble sleeping**			**Trouble sleeping**		
	**3–6 h/day**	**7–8 h/day**	**>8 h/day**	**3–6 h/day**	**7–8 h/day**	**>8 h/day**
**Hypertension**						
Person-year	18386.8	26811.7	4255.6	14071.0	8497.8	1345.4
No. of cases	327	663	202	333	179	45
HR, model 1	0.97 (0.83, 1.14)	1.00	1.42 (1.20, 1.67)	1.29 (1.05, 1.58)	1.00	1.57 (1.04, 2.38)
HR, model 2	0.85 (0.71, 1.01)	1.00	1.23 (1.04, 1.46)	1.20 (0.95, 1.52)	1.00	1.44 (0.92, 2.25)

**High cholesterol**						
Person-year	15817.8	27040.3	3772.4	12038.3	8522.5	1216.3
No. of cases	210	471	134	241	147	32
HR, model 1	1.10 (0.89, 1.37)	1.00	1.33 (1.08, 1.65)	1.41 (1.09, 1.84)	1.00	1.68 (1.10, 2.56)
HR, model 2	0.95 (0.75, 1.19)	1.00	1.15 (0.92, 1.45)	1.20 (0.92, 1.58)	1.00	1.47 (0.95, 2.27)

**Diabetes**						
Person-year	8698.2	13286.8	2304	6425.2	3696.4	727.8
No. of cases	199	389	123	187	91	31
HR, model 1	0.92 (0.71, 1.18)	1.00	**1.44 (1.10, 1.87)**	**1.53 (1.21, 1.94)**	1.00	**1.69 (1.05, 2.71)**
HR, model 2	0.87 (0.67, 1.13)	1.00	**1.38 (1.07, 1.78)**	**1.46 (1.13, 1.90)**	1.00	**1.62 (1.00, 2.65)**

**Chronic renal failure**						
Person-year	13696.5	22765.9	3431.4	6161.4	4303.3	772.3
No. of cases	283	621	181	196	130	29
HR, model 1	1.02 (0.85, 1.24)	1.00	1.49 (1.21, 1.85)	1.34 (1.08, 1.67)	1.00	1.38 (0.83, 2.29)
HR, model 2	0.92 (0.73, 1.15)	1.00	1.35 (1.06, 1.72)	1.22 (0.96, 1.57)	1.00	1.35 (0.76, 2.39)

Model 1 was the Cox proportional hazard model adjusted for age and sex.

Model 2 was based on model 1 and further adjusted for ethnicity, body mass index, income, education level, smoking status, alcohol intake, moderate physical activity, hypertension, high cholesterol, diabetes, and heart disease.

P-interaction between sleep hour and trouble sleeping = 0.123 for hypertension, 0.116 for high cholesterol, **0.042 for diabetes**, and 0.506 for chronic renal failure.

## 4. Discussion

In the current study of participants with chronic NCD from NHANES, sleep duration of over 8 h/day was significantly associated with higher risks of all-cause mortality and mortality from heart diseases. People with NCD of over 8 h/day sleep duration were associated with 23% to 44% and 55% to 134% higher risks of all-cause mortality and heart disease mortality than those of 7–8 h/day, respectively. We detected a nonlinear association between sleep duration and all-cause mortality. In addition, the adverse association between the long sleep duration and all-cause mortality was stronger in older patients with hypertension, and patients with diabetes having trouble sleeping. Our data may offer valuable evidence on tailor healthy sleep recommendations against the long-term mortality risk in people with chronic NCD.

Accumulating epidemiological studies have indicated the association between long sleep duration and elevated risks of all-cause and cause-specific mortality, with only a few studies conducted among people with chronic disease [30–32]. Previous findings detected a J-shaped relationship between sleep duration and all-cause mortality risk in people with T2DM, where those who slept less or more than 7 h/day had higher risks of all-cause and condition-specific mortality [33, 34]. The Korea National Health and Nutrition Examination Survey found that long sleep duration was significantly associated with a 65% higher risk of all-cause mortality (HR, 95%CI = 1.65, 1.29–2.11) in comparison to sleep duration of 7 h/day in patients with diabetes, where the medical conditions of CKD and cancer were identified as modifiers [33]. According to National Health Interview Survey in the U.S., it was suggested that compared with the 7 h/day group, both shorter and longer sleep durations were associated with a higher risk of all-cause mortality (≤5 h/day, HR = 1.24, 1.09–1.40; 6 h/day, HR = 1.13, 1.01–1.28; 8 h/day, HR = 1.17, 1.06–1.30; ≥10 h/day, HR = 1.83, 1.61–2.08) in patients with diabetes [34]. Similar associations were also detected for mortality from CVD, cancer, CKD, Alzheimer’s disease, and chronic lower respiratory diseases [34]. Besides, long sleep duration (>8 h/day v.s. 6–8 h/day) was associated with increased risks of death and poor health-related quality of life in Korean adults with CKD [22], whereas in contrast, a prospective cohort study from the U.S. showed that both sleep duration and sleep efficiency were not associated with mortality in individuals with advanced CKD, but it only consisted of 180 patients [21]. The difference in patient characteristics, the ascertainment of disease, the course of illness, and the covariate adjustment might result in the divergence of the findings.

Previous studies have linked long sleep duration to higher systemic inflammatory responses, where the inflammatory cytokines including C-reactive protein (CRP) and interleukin-6 (IL-6) were upregulated in people with longer sleep duration [35–37]. In patients with chronic NCD, the inflammation could accelerate the progressive condition of NCD, thus increasing mortality risk. In addition, long sleep duration (>8 h/day) significantly increased the risks of high blood pressure, dyslipidemia, high fasting blood glucose, and high waist circumference, potentially leading to the deterioration of NCD clinical outcomes [38, 39]. Long sleep duration may also indicate other risk factors for survival in patients with chronic disease, such as a sedentary lifestyle, low socioeconomic status, depression, cognitive decline, and the existence of comorbidities [40–43].

In the subgroup analysis, we found the older (≥65 years) with sleep duration over 8 h/day had a higher risk of mortality than the young (<65 years) patients with hypertension. Older people may experience more severe inflammatory responses, and higher rates of frailty, multimorbidity, and polypharmacy, referring to a higher risk of mortality. It is imperative to understand that the long sleep duration that accompanies aging may worsen the health of an older population, especially older adults with chronic disease. Interestingly, coexisting trouble sleeping and long sleep duration exerted a joint adverse effect on mortality risk in patients with diabetes. A previous meta-analysis showed that compared to normal sleep duration, insomnia disorder with short sleep was associated with a higher risk of hypertension and T2DM [44]. Poor sleep measures, including chronotype, duration, insomnia complaints, snoring, and daytime sleepiness, were found to be adversely associated with CVD-free life expectancy, especially pronounced in individuals with sleep-related breathing disorders [45]. Trouble sleeping contributes to disturbances in the biological clock and the biological rhythm of insulin secretion by pancreatic islet cells. Meanwhile, the sympathetic nervous system may be activated by trouble sleeping, leading to high levels of growth hormone and cortisol, reduced glucose tolerance, a decrease in insulin sensitivity, and incidence of insulin resistance [46]. The hormonal disorders also resulted in decreased leptin and elevated ghrelin levels, enhanced food intake, altered appetite and glucose metabolism, weight gain and obesity [46–48]. Therefore, sleep time and trouble sleeping may be valuable indicators and help to identify patients with diabetes at higher risk of poorer health consequences, even mortality.

We used nationally representative data and a prospective cohort design to illustrate the association between sleep factors and the risk of mortality in patients with chronic NCD. However, several limitations should be acknowledged. First, the assessment of sleep information was self-report, possibly leading to misreporting and measurement error. However, this measurement error was inclined to be non-differential in the prospective study and resulted in associations being attenuated towards the null. Besides, the sleep assessment was based on the baseline cycle; hence, misclassifications of sleep status were possible. Another limitation of current study in patients with NCD was the heterogeneity of participants in terms of disease severity and duration. Additionally, the nature of the trouble sleeping and sleep disorder reported in the NHANES dataset is self-reported, which were not well-defined and distinguished, such as obstructive sleep apnea (OSA), sleep quality (sleep deprivation, sleep duration and insomnia), or combinations of sleep problems. Last, although a multitude of conventional confounding factors both associated with sleep and mortality have been considered, residual confounding (such as medication use and psychological conditions) cannot be excluded and remained a possible explanation for the observed associations.

In summary, our study indicates that long sleep duration (>8 h/day) was associated with higher risks of all-cause mortality and mortality from heart diseases in patients with chronic NCD, including hypertension, high cholesterol, diabetes, and chronic renal failure. Our findings have important public health implications and highlight that improving sleep behaviors may decrease the risk of premature deaths and help in the tertiary prevention of NCD.

## Abbreviations

NHANES: National Health and Nutrition Examination Survey
NCD: Noncommunicable disease
CVD: Cardiovascular disease
CKD: chronic kidney disease
T2DM: type 2 diabetes
BMI: body mass index
GFR: glomerular filtration rate

## Supplementary information

**Additional file 1:** **Supplementary Table 1.** Sex-specific hazard ratios (HRs) and 95% confidence intervals (CIs) for all-cause mortality according to sleep duration. **Supplementary Table 2.** Hazard ratios (HRs) and 95% confidence intervals (CIs) for all-cause mortality according to sleep duration, stratified by sleep disorder. **Supplementary Table 3.** Sensitivity analysis for the hazard ratios (HRs) of all-cause mortality in relation to different cutoff points of sleep duration in patients with at least one chronic NCD. **Supplementary Table 4.** Sensitivity analysis for the hazard ratios (HRs) of all-cause mortality in relation to sleep duration in patients with NCD (excluding patients with mortality during the first 3 years of follow-up). **Supplementary Table 5.** Sensitivity analysis for the hazard ratios (HRs) of all-cause mortality in relation to sleep duration in participants without any NCD. **Supplementary Figure 1.** Dose–response association of sleep duration with all-cause mortality in participants with hypertension. **Supplementary Figure 2.** Dose–response association of sleep duration with all-cause mortality in participants with high cholesterol. **Supplementary Figure 3.** Dose–response association of sleep duration with all-cause mortality in participants with diabetes. **Supplementary Figure 4.** Dose–response association of sleep duration with all-cause mortality in participants with chronic kidney disease. **Supplementary Figure 5.** Dose–response association of sleep duration with all-cause mortality in participants without any NCD.

## Declarations

### Availability of data and materials

The datasets can be found in online repositories. At: https://www.cdc.gov/nchs/nhanes/index.htm.
