# Supplementary material for: Sleep duration and mortality in patients with chronic noncommunicable disease: a population-based cohort study
Source: Environ Health Prev Med. 2024 Feb 29;29:9. doi: 10.1265/ehpm.23-00249 (PMC10937247; doi:10.1265/ehpm.23-00249)
Supplement: Supplementary file 1 — Additional file 1: Supplementary Table 1. Sex-specific hazard ratios (HRs) and 95% confidence intervals (CIs) for all-cause mortality according to sleep duration. Supplementary Table 2. Hazard ratios (HRs) and 95% confidence intervals (CIs) for all-cause mortality according to sleep duration, stratified by sleep disorder. Supplementary Table 3. Sensitivity analysis for the hazard ratios (HRs) of all-cause mortality in relation to different cutoff points of sleep duration in patients with at least one chronic NCD. Supplementary Table 4. Sensitivity analysis for the hazard ratios (HRs) of all-cause mortality in relation to sleep duration in patients with NCD (excluding patients with mortality during the first 3 years of follow-up). Supplementary Table 5. Sensitivity analysis for the hazard ratios (HRs) of all-cause mortality in relation to sleep duration in participants without any NCD. Supplementary Figure 1. Dose–response association of sleep duration with all-cause mortality in participants with hypertension. Supplementary Figure 2. Dose–response association of sleep duration with all-cause mortality in participants with high cholesterol. Supplementary Figure 3. Dose–response association of sleep duration with all-cause mortality in participants with diabetes. Supplementary Figure 4. Dose–response association of sleep duration with all-cause mortality in participants with chronic kidney disease. Supplementary Figure 5. Dose–response association of sleep duration with all-cause mortality in participants without any NCD. [file ehpm-29-009-s001.docx]

Appendices

Supplementary Files

Supplementary Table 1. Sex-specific hazard ratios (HRs) and 95% confidence intervals (CIs) for all-cause mortality according to sleep duration.

Supplementary Table 2. Hazard ratios (HRs) and 95% confidence intervals (CIs) for all-cause mortality according to sleep duration, stratified by sleep disorder.

Supplementary Table 3. Sensitivity analysis for the hazard ratios (HRs) of all-cause mortality in relation to different cutoff points of sleep duration in patients with at least one chronic NCD.

Supplementary Table 4. Sensitivity analysis for the hazard ratios (HRs) of all-cause mortality in relation to sleep duration in patients with NCD (excluding patients with mortality during the first 3 years of follow-up).

Supplementary Table 5. Sensitivity analysis for the hazard ratios (HRs) of all-cause mortality in relation to sleep duration in participants without any NCD.

Supplementary Figure 1. Dose–response association of sleep duration with all-cause mortality in participants with hypertension.

Supplementary Figure 2. Dose–response association of sleep duration with all-cause mortality in participants with high cholesterol.

Supplementary Figure 3. Dose–response association of sleep duration with all-cause mortality in participants with diabetes.

Supplementary Figure 4. Dose–response association of sleep duration with all-cause mortality in participants with chronic kidney disease.

Supplementary Figure 5. Dose–response association of sleep duration with all-cause mortality in participants without any NCD.

Supplementary Table 1. Sex-specific hazard ratios (HRs) and 95% confidence intervals (CIs) for all-cause mortality according to sleep duration.

|  | Men | | | Women | | |
| --- | --- | --- | --- | --- | --- | --- |
|  | 3–6 h/day | 7–8 h/day | >8 h/day | 3–6 h/day | 7–8 h/day | >8 h/day |
| **Hypertension** |  |  |  |  |  |  |
| Person-year | 15452.3 | 16357.7 | 2444.7 | 17005.6 | 18951.8 | 3156.3 |
| No. of cases | 322 | 438 | 121 | 338 | 404 | 126 |
| HR, model 1 | 1.19 (0.93, 1.52) | 1.00 | 1.43 (1.05, 1.95) | 1.06 (0.91, 1.23) | 1.00 | 1.51 (1.22, 1.87) |
| HR, model 2 | 1.00 (0.77, 1.28) | 1.00 | 1.21 (0.91, 1.59) | 1.00 (0.84, 1.19) | 1.00 | 1.33 (1.06, 1.68) |
| HR, model 3 | 0.99 (0.78, 1.27) | 1.00 | 1.21 (0.92, 1.58) | 0.96 (0.82, 1.13) | 1.00 | 1.35 (1.07, 1.69) |
|  |  |  |  |  |  |  |
| **High cholesterol** |  |  |  |  |  |  |
| Person-year | 13842.5 | 17502.2 | 2081.8 | 14013.6 | 18060.6 | 2906.8 |
| No. of cases | 235 | 333 | 82 | 216 | 285 | 84 |
| HR, model 1 | 1.44 (1.14, 1.82) | 1.00 | 1.36 (0.95, 1.96) | 1.08 (0.88, 1.31) | 1.00 | 1.44 (1.09, 1.89) |
| HR, model 2 | 1.12 (0.86, 1.47) | 1.00 | 1.22 (0.82, 1.81) | 0.99 (0.80, 1.23) | 1.00 | 1.22 (0.93, 1.61) |
| HR, model 3 | 1.12 (0.85, 1.48) | 1.00 | 1.21 (0.81, 1.81) | 0.99 (0.80, 1.24) | 1.00 | 1.22 (0.92, 1.61) |
|  |  |  |  |  |  |  |
| **Diabetes** |  |  |  |  |  |  |
| Person-year | 7617.5 | 8746.9 | 1360.2 | 7506.0 | 8236.3 | 1671.6 |
| No. of cases | 226 | 268 | 71 | 160 | 212 | 83 |
| HR, model 1 | 1.30 (0.99, 1.70) | 1.00 | 1.49 (0.98, 2.25) | 0.96 (0.73, 1.27) | 1.00 | 1.47 (1.08, 2.01) |
| HR, model 2 | 1.22 (0.91, 1.63) | 1.00 | 1.28 (0.81, 2.00) | 0.92 (0.69, 1.24) | 1.00 | 1.48 (1.09, 2.00) |
| HR, model 3 | 1.28 (0.97, 1.67) | 1.00 | 1.26 (0.81, 1.97) | 0.89 (0.67, 1.18) | 1.00 | 1.50 (1.10, 2.04) |
|  |  |  |  |  |  |  |
| **Chronic renal failure** |  |  |  |  |  |  |
| Person-year | 14288.4 | 19730.1 | 2761.6 | 5569.5 | 7339.1 | 1442.1 |
| No. of cases | 342 | 532 | 137 | 137 | 219 | 73 |
| HR, model 1 | 1.25 (1.06, 1.47) | 1.00 | 1.46 (1.09, 1.96) | 0.9 (0.71, 1.14) | 1.00 | 1.47 (1.07, 2.01) |
| HR, model 2 | 1.03 (0.84, 1.27) | 1.00 | 1.31 (0.98, 1.75) | 0.94 (0.70, 1.24) | 1.00 | 1.43 (0.96, 2.13) |
| HR, model 3 | 1.04 (0.85, 1.26) | 1.00 | 1.32 (0.99, 1.75) | 0.96 (0.72, 1.27) | 1.00 | 1.47 (0.99, 2.18) |
|  |  |  |  |  |  |  |

Model 1 was the Cox proportional hazard model adjusted for age.

Model 2 was based on model 1 and further adjusted for ethnicity, body mass index, income, education level, smoking status, alcohol intake, moderate physical activity, hypertension, high cholesterol, diabetes, and heart disease.

Model 3 was based on model 2 and additionally adjusted for trouble sleeping and sleep disorder.

P-interaction between sleep hour and sex = 0.701 for hypertension, 0.634 for high cholesterol, 0.647 for diabetes, and 0.959 for chronic renal failure.

Supplementary Table 2. Hazard ratios (HRs) and 95% confidence intervals (CIs) for all-cause mortality according to sleep duration, stratified by sleep disorder.

|  | No sleep disorder | | | Sleep disorder | | |
| --- | --- | --- | --- | --- | --- | --- |
|  | 3–6 h/day | 7–8 h/day | >8 h/day | 3–6 h/day | 7–8 h/day | >8 h/day |
| **Hypertension** |  |  |  |  |  |  |
| Person-year | 18386.8 | 26811.7 | 4255.6 | 5022.2 | 3552.1 | 604.7 |
| No. of cases | 327 | 663 | 202 | 108 | 83 | 24 |
| HR, model 1 | 0.97 (0.83, 1.14) | 1.00 | 1.42 (1.20, 1.67) | 1.22 (0.86, 1.73) | 1.00 | 1.86 (1.11, 3.11) |
| HR, model 2 | 0.85 (0.71, 1.01) | 1.00 | 1.23 (1.04, 1.46) | 1.18 (0.8, 1.74) | 1.00 | 1.48 (0.88, 2.49) |
|  |  |  |  |  |  |  |
| **High cholesterol** |  |  |  |  |  |  |
| Person-year | 15817.8 | 27040.3 | 3772.4 | 4085.8 | 3238.7 | 527.4 |
| No. of cases | 210 | 471 | 134 | 84 | 63 | 18 |
| HR, model 1 | 1.10 (0.89, 1.37) | 1.00 | 1.33 (1.08, 1.65) | 1.45 (0.9, 2.33) | 1.00 | 1.59 (0.92, 2.77) |
| HR, model 2 | 0.95 (0.75, 1.19) | 1.00 | 1.15 (0.92, 1.45) | 1.37 (0.84, 2.24) | 1.00 | 1.17 (0.60, 2.29) |
|  |  |  |  |  |  |  |
| **Diabetes** |  |  |  |  |  |  |
| Person-year | 8698.2 | 13286.8 | 2304 | 2449.2 | 1828.0 | 330.4 |
| No. of cases | 199 | 389 | 123 | 72 | 47 | 17 |
| HR, model 1 | 0.92 (0.71, 1.18) | 1.00 | 1.44 (1.10, 1.87) | 1.7 (0.97, 2.95) | 1.00 | 2.29 (1.12, 4.68) |
| HR, model 2 | 0.87 (0.67, 1.13) | 1.00 | 1.38 (1.07, 1.78) | 1.49 (0.86, 2.58) | 1.00 | 2.06 (0.97, 4.35) |
|  |  |  |  |  |  |  |
| **Chronic renal failure** |  |  |  |  |  |  |
| Person-year | 13696.5 | 22765.9 | 3431.4 | 2288.6 | 1763.8 | 336.5 |
| No. of cases | 283 | 621 | 181 | 67 | 60 | 17 |
| HR, model 1 | 1.02 (0.85, 1.24) | 1.00 | 1.49 (1.21, 1.85) | 1.35 (0.92, 2.00) | 1.00 | 2.05 (1.12, 3.73) |
| HR, model 2 | 0.92 (0.73, 1.15) | 1.00 | 1.35 (1.06, 1.72) | 1.23 (0.77, 1.96) | 1.00 | 1.83 (1.05, 3.20) |
|  |  |  |  |  |  |  |

Model 1: adjusted for sex and age.

Model 2 was based on model 1 and further adjusted for ethnicity, body mass index, income, education level, smoking status, alcohol intake, moderate physical activity, hypertension, high cholesterol, diabetes, and heart disease.

P-interaction between sleep hour and sleep disorder = 0.255 for hypertension, 0.667 for high cholesterol, 0.153 for diabetes, and 0.398 for chronic renal failure.

Supplementary Table 3. Sensitivity analysis for the hazard ratios (HRs) of all-cause mortality in relation to different cutoff points of sleep duration in patients with at least one chronic NCD.

|  | Sleep hours | | | |
| --- | --- | --- | --- | --- |
|  | <6 h/day | 6 h/day | 7–8 h/day | >8 h/day |
| Person-year | 22656.0 | 32525.4 | 68587.2 | 9724.7 |
| No. of cases | 427 | 487 | 1243 | 357 |
| HR, model 1 | 1.39 (1.21, 1.60) | 1.00 | 0.96 (0.85, 1.08) | 1.53 (1.31, 1.80) |
| HR, model 2 | 1.15 (0.99, 1.34) | 1.00 | 0.99 (0.87, 1.14) | 1.38 (1.19, 1.61) |
| HR, model 3 | 1.14 (0.98, 1.32) | 1.00 | 1.00 (0.88, 1.14) | 1.40 (1.20, 1.62) |

NCD, noncommunicable disease

Model 1 was the Cox proportional hazard model adjusted for age and sex.

Model 2 was based on model 1 and further adjusted for ethnicity, body mass index, income, education level, smoking status, alcohol intake, moderate physical activity, hypertension, high cholesterol, diabetes, and heart disease.

Model 3 was based on model 2 and additionally adjusted for trouble sleeping and sleep disorder.

Supplementary Table 4. Sensitivity analysis for the hazard ratios (HRs) of all-cause mortality in relation to sleep duration in patients with NCD (excluding patients with mortality during the first 3 years of follow-up).

|  | Sleep hours | | |
| --- | --- | --- | --- |
|  | 3–6 h/day | 7–8 h/day | >8 h/day |
| **Hypertension (n=7923)** |  |  |  |
| Person-year | 32171.9 | 34981.7 | 5479.8 |
| No. of cases | 525 | 684 | 188 |
| HR, model 1 | 1.07 (0.92, 1.25) | 1.00 | 1.43 (1.19, 1.72) |
| HR, model 2 | 0.96 (0.81, 1.14) | 1.00 | 1.26 (1.04, 1.51) |
| HR, model 3 | 0.94 (0.80, 1.12) | 1.00 | 1.26 (1.05, 1.51) |
|  |  |  |  |
| **High cholesterol (n=7323)** |  |  |  |
| Person-year | 27682.7 | 35343.4 | 4911.3 |
| No. of cases | 370 | 513 | 128 |
| HR, model 1 | 1.22 (1.01, 1.47) | 1.00 | 1.37 (1.11, 1.70) |
| HR, model 2 | 1.05 (0.85, 1.3) | 1.00 | 1.22 (0.98, 1.50) |
| HR, model 3 | 1.05 (0.84, 1.3) | 1.00 | 1.21 (0.98, 1.51) |
|  |  |  |  |
| **Diabetes (n=3862)** |  |  |  |
| Person-year | 14958.8 | 16809.4 | 2955.9 |
| No. of cases | 306 | 396 | 115 |
| HR, model 1 | 1.04 (0.83, 1.32) | 1.00 | 1.34 (1.01, 1.77) |
| HR, model 2 | 0.98 (0.76, 1.25) | 1.00 | 1.29 (0.99, 1.68) |
| HR, model 3 | 0.99 (0.78, 1.25) | 1.00 | 1.29 (0.99, 1.68) |
|  |  |  |  |
| **Chronic renal failure (n=5477)** |  |  |  |
| Person-year | 19608.9 | 26737.6 | 4080.0 |
| No. of cases | 361 | 591 | 150 |
| HR, model 1 | 1.05 (0.9, 1.24) | 1.00 | 1.41 (1.11, 1.79) |
| HR, model 2 | 0.96 (0.78, 1.17) | 1.00 | 1.29 (0.98, 1.70) |
| HR, model 3 | 0.97 (0.79, 1.19) | 1.00 | 1.29 (0.98, 1.69) |
|  |  |  |  |

NCD, noncommunicable disease.

Model 1 was the Cox proportional hazard model adjusted for age and sex.

Model 2 was based on model 1 and further adjusted for ethnicity, body mass index, income, education level, smoking status, alcohol intake, moderate physical activity, hypertension, high cholesterol, diabetes, and heart disease.

Model 3 was based on model 2 and additionally adjusted for trouble sleeping and sleep disorder.

Supplementary Table 5. Sensitivity analysis for the hazard ratios (HRs) of all-cause mortality in relation to sleep duration in participants without any NCD.

|  | Sleep hours | | |
| --- | --- | --- | --- |
|  | 3–6 h/day (n=3900) | 7–8 h/day (n=5972) | >8 h/day (n=709) |
| Person-year | 38415.2 | 59312.8 | 6971.1 |
| No. of cases | 133 | 177 | 43 |
| HR, model 1 | 1.50 (1.13, 1.99) | 1.00 | 2.09 (1.35, 3.23) |
| HR, model 2 | 1.38 (1.02, 1.86) | 1.00 | 1.64 (1.03, 2.61) |
| HR, model 3 | 1.31 (0.97, 1.77) | 1.00 | 1.60 (0.99, 2.59) |

NCD, noncommunicable disease

Model 1 was the Cox proportional hazard model adjusted for age and sex.

Model 2 was based on model 1 and further adjusted for ethnicity, body mass index, income, education level, smoking status, alcohol intake, moderate physical activity, hypertension, high cholesterol, diabetes, and heart disease.

Model 3 was based on model 2 and additionally adjusted for trouble sleeping and sleep disorder.


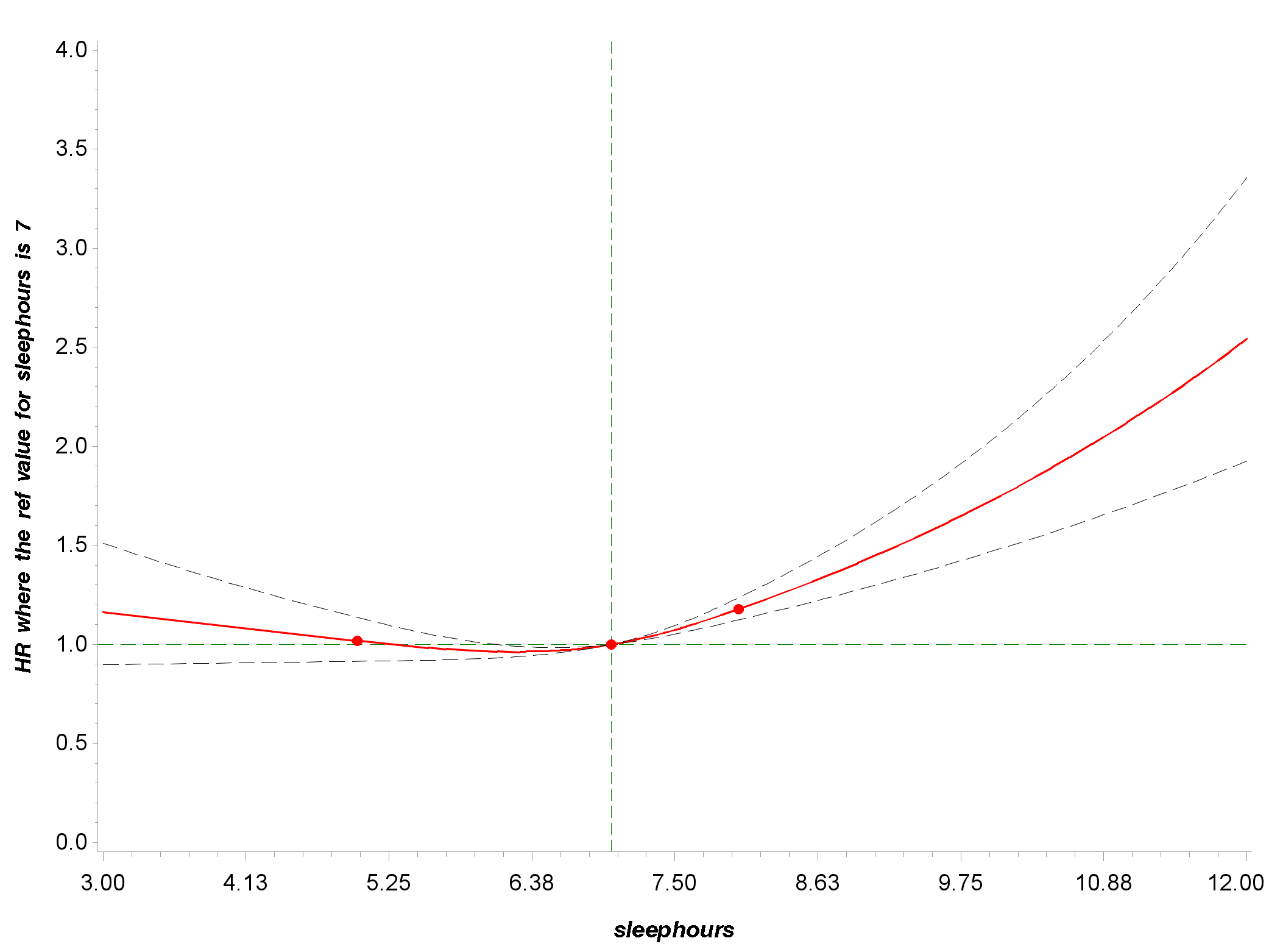


Supplementary Figure 1. Dose–response association of sleep duration with all-cause mortality in participants with hypertension.

P overall association < 0.001; P nonlinear association < 0.001.

Restricted cubic splines were constructed with four knots located at the 10th, 50th, and 95th percentiles of the exposure. Adjusted hazard ratios (95% CI) were calculated with adjustment for age, sex, ethnicity, body mass index, income, education level, smoking status, alcohol intake, moderate physical activity, high cholesterol, diabetes, and heart disease.


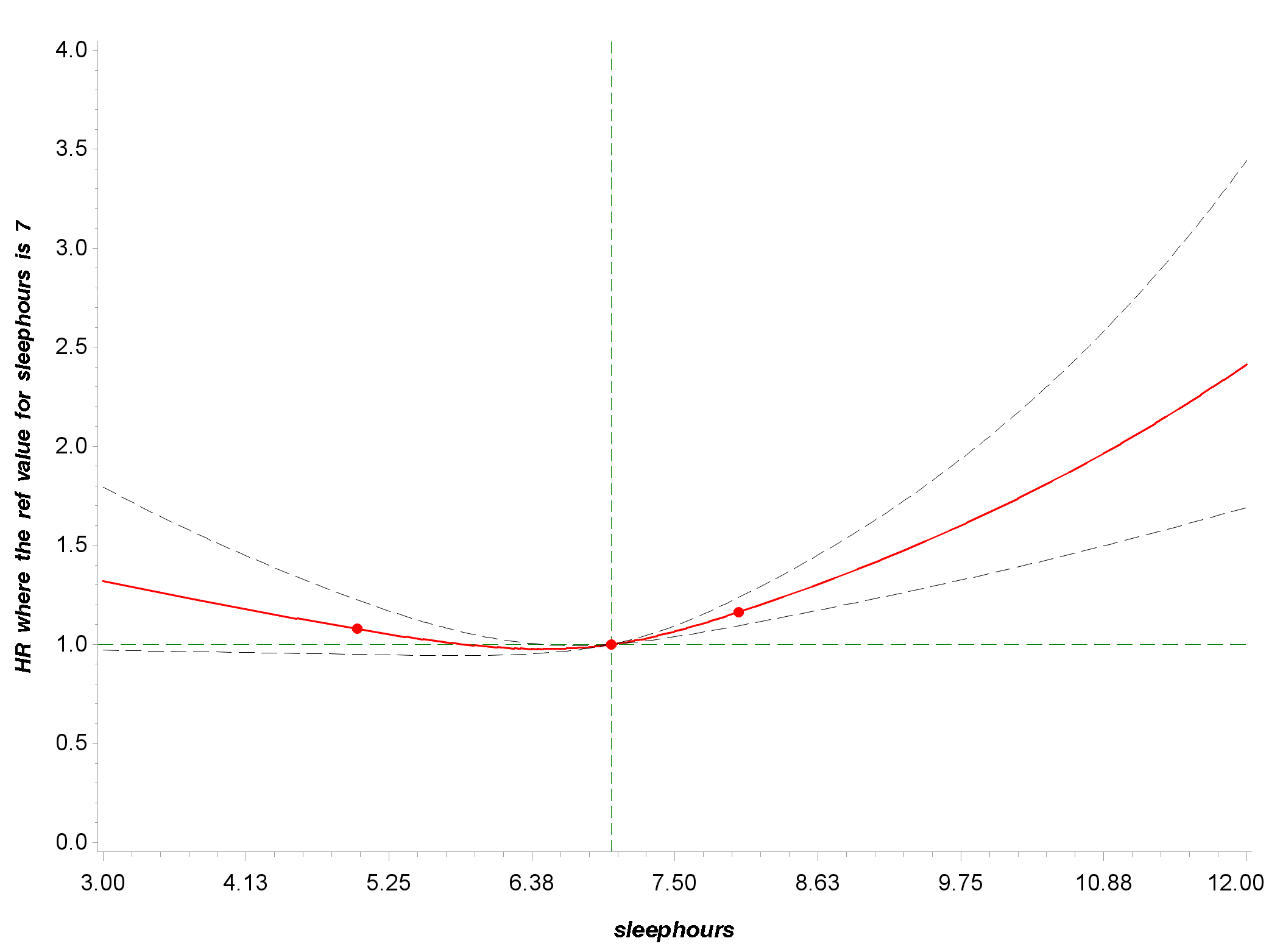


Supplementary Figure 2. Dose–response association of sleep duration with all-cause mortality in participants with high cholesterol.

P overall association < 0.001; P nonlinear association < 0.001.

Restricted cubic splines were constructed with four knots located at the 10th, 50th, and 95th percentiles of the exposure. Adjusted hazard ratios (95% CI) were calculated with adjustment for age, sex, ethnicity, body mass index, income, education level, smoking status, alcohol intake, moderate physical activity, hypertension, diabetes, and heart disease.


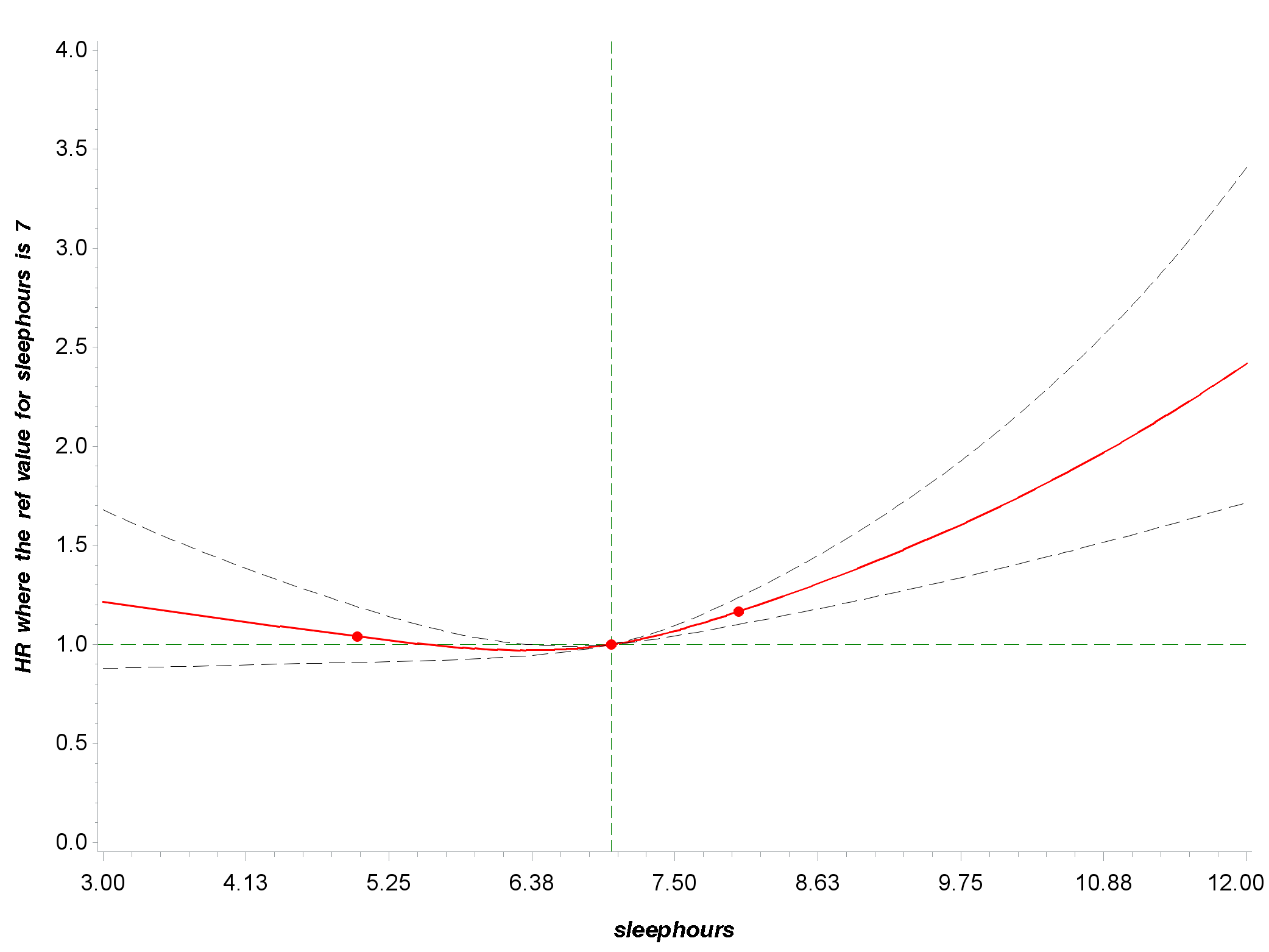


Supplementary Figure 3. Dose–response association of sleep duration with all-cause mortality in participants with diabetes.

P overall association < 0.001; P nonlinear association < 0.001.

Restricted cubic splines were constructed with four knots located at the 10th, 50th, and 95th percentiles of the exposure. Adjusted hazard ratios (95% CI) were calculated with adjustment for age, sex, ethnicity, body mass index, income, education level, smoking status, alcohol intake, moderate physical activity, hypertension, high cholesterol, and heart disease.


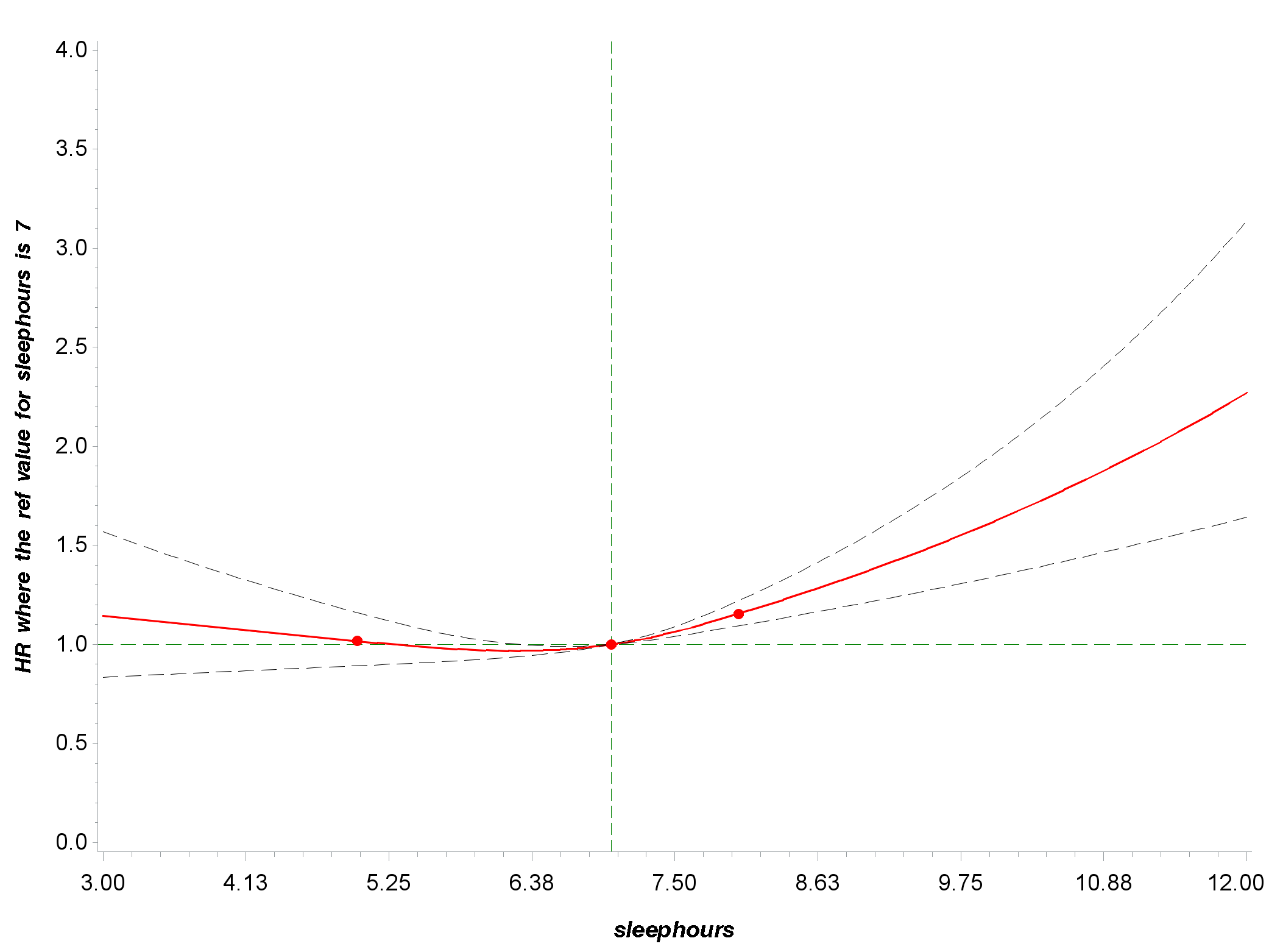


Supplementary Figure 4. Dose–response association of sleep duration with all-cause mortality in participants with chronic renal failure.

P overall association < 0.001; P nonlinear association = 0.001.

Restricted cubic splines were constructed with four knots located at the 10th, 50th, and 95th percentiles of the exposure. Adjusted hazard ratios (95% CI) were calculated with adjustment for age, sex, ethnicity, body mass index, income, education level, smoking status, alcohol intake, moderate physical activity, hypertension, high cholesterol, diabetes, and heart disease.


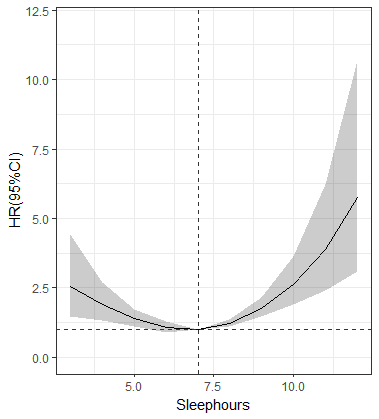


Supplementary Figure 5. Dose–response association of sleep duration with all-cause mortality in participants without any NCD.

P overall association < 0.001; P nonlinear association < 0.001.

Restricted cubic splines were constructed with four knots located at the 10th, 50th, and 95th percentiles of the exposure. Adjusted hazard ratios (95% CI) were calculated with adjustment for age, sex, ethnicity, body mass index, income, education level, smoking status, alcohol intake, and moderate physical activity.
